# Supplementary material for: The Evolution and Characterization of the RNA Interference Pathways in Lophotrochozoa
Source: Genome Biol Evol. 2024 May 7;16(5):evae098. doi: 10.1093/gbe/evae098 (PMC11114477; doi:10.1093/gbe/evae098)
Supplement: evae098_Supplementary_Data [file evae098_supplementary_data.zip › Supplementary_materials.zip!/selected_miRNAs/NoGe_selected_miRNAs/mirdeep_pdf_files/NMRB01001262.1_Notospermus_geniculatus_strain_Ushimado_scaffold1262,_whole_genome_shotgun_sequence_26851.pdf]

Provisional ID	:	NMRB01001262.1_Notospermus_geniculatus_strain_Ushimado_scaffold1262,_whole_genome_shotgun_sequence_26851
Score total	:	107269.3
Score for star read(s)	:	3.9
Score for read counts	:	107260.7
Score for mfe	:	1.7
Score for randfold	:	0
Score for cons. seed	:	3
Total read count	:	210399
Mature read count	:	210206
Loop read count	:	0
Star read count	:	193

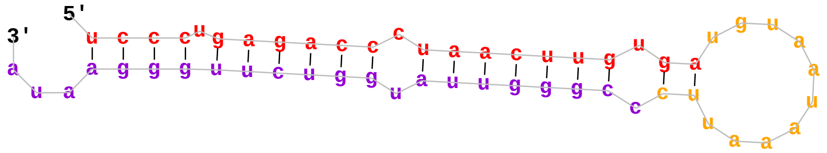

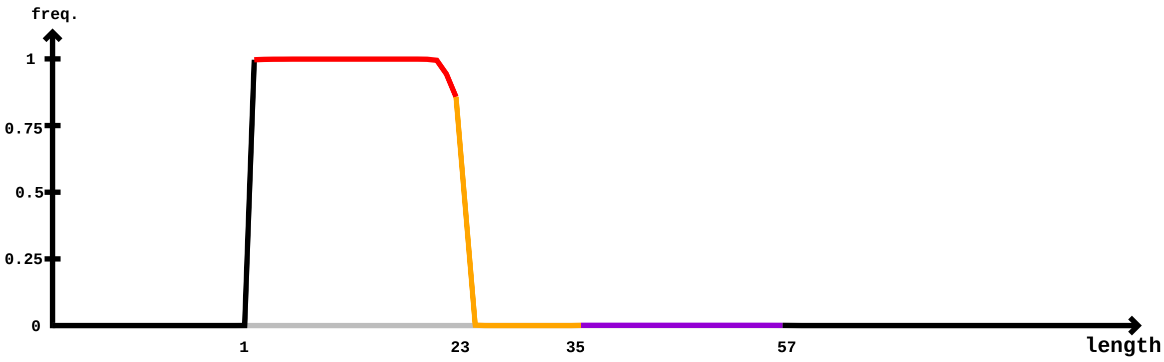

Mature Star

```
5'- cguggaggcuuccuccccuucccugagacccuaacuugugauguaauaaaauucccggguuauggucuugggaauagggugguugcuauggcaacggaauacgacuaucacc -3'   obs
    cguggaggcuuccuccccuucccugagacccuaacuugugauguaauaaaauucccggguuauggucuugggaauagggugguugcuauggcaacggaauacgacuaucacc        exp
    .((.(.(((..((.(((.(((((.((((((.(((((((.((..........)).))))))).)))))))))))..))).))..))).).))..((.....))..........   reads   mm   sample
    ...................ucccugagacccuaacuA...........................................................................   1       1    Mm1
    ...................ucccugagacccuaacuu...........................................................................   28      0    Mm1
    ...................ucccugagacUcuaacuug..........................................................................   1       1    Mm1
    ...................ucccugagacccuaacuuU..........................................................................   8       1    Mm1
    ...................ucccugagacccuaacAug..........................................................................   1       1    Mm1
    ...................ucccugagacccuaacuuA..........................................................................   10      1    Mm1
    ...................ucccugagacccuaacuug..........................................................................   53      0    Mm1
    ...................ucccugagacccuaacuGg..........................................................................   1       1    Mm1
    ...................ucccugagacAcuaacuug..........................................................................   1       1    Mm1
    ...................ucccugagacccuaacuCgu.........................................................................   3       1    Mm1
    ...................ucccugagacUcuaacuugu.........................................................................   1       1    Mm1
    ...................ucccugagacccuaacuuUu.........................................................................   1       1    Mm1
    ...................ucccugagacccuaacCugu.........................................................................   4       1    Mm1
    ...................ucccugagacccuaacuGgu.........................................................................   4       1    Mm1
    ...................Ncccugagacccuaacuugu.........................................................................   1       1    Mm1
    ...................ucccugagacccuaacuAgu.........................................................................   5       1    Mm1
    ...................ucccugagacGcuaacuugu.........................................................................   1       1    Mm1
    ...................ucccugagacccuaacuugu.........................................................................   775     0    Mm1
    ...................ucAcugagacccuaacuugu.........................................................................   1       1    Mm1
    ...................ucccugagacccuaacuugAg........................................................................   1       1    Mm1
    ...................uUccugagacccuaacuugug........................................................................   1       1    Mm1
    ...................ucccugagaAccuaacuugug........................................................................   2       1    Mm1
    ...................ucccugagacccuaacuuguU........................................................................   9       1    Mm1
    ...................ucccugagacUcuaacuugug........................................................................   2       1    Mm1
    ...................ucccugagacccuaacAugug........................................................................   2       1    Mm1
    ...................uAccugagacccuaacuugug........................................................................   1       1    Mm1
    ...................uGccugagacccuaacuugug........................................................................   1       1    Mm1
    ...................ucccugagacAcuaacuugug........................................................................   2       1    Mm1
    ...................ucccugagacccuaacuAgug........................................................................   14      1    Mm1
    ...................ucGcugagacccuaacuugug........................................................................   1       1    Mm1
    ...................ucccugagacGcuaacuugug........................................................................   2       1    Mm1
    ...................ucccugagacccuCacuugug........................................................................   3       1    Mm1
    ...................ucccCgagacccuaacuugug........................................................................   1       1    Mm1
```

Mature   Star

```
cguggaggcuuccuccccuucccugagacccuaacuugugauguaauaaauucccggguuauggucuugggaauagggugguugcuauggcaacggaauacgacuaucacc
...................ucccugagacccuaacuugGg.......................................................................   1      1    Mm1
...................ucccugagacccuaacuugCg.......................................................................   1      1    Mm1
...................ucccugagacccuaacuCgug.......................................................................   17     1    Mm1
...................ucccugagacccuGacuugug.......................................................................   2      1    Mm1
...................ucccugagacccuaacuGgug.......................................................................   12     1    Mm1
...................Ncccugagacccuaacuugug.......................................................................   1      1    Mm1
...................ucccugagacccuaacuugug.......................................................................   2496   0    Mm1
...................ucccugagUcccuaacuugug.......................................................................   2      1    Mm1
...................ucccugagacccuaacGugug.......................................................................   7      1    Mm1
...................ucccugagacccuaacCugug.......................................................................   10     1    Mm1
...................ucccugaUacccuaacuugug.......................................................................   1      1    Mm1
...................Ccccugagacccuaacuuguga......................................................................   2      1    Mm1
...................ucNcugagacccuaacuuguga......................................................................   1      1    Mm1
...................uUccugagacccuaacuuguga......................................................................   4      1    Mm1
...................ucccugagacccuaacuugugG......................................................................   721    1    Mm1
...................Gcccugagacccuaacuuguga......................................................................   2      1    Mm1
...................ucccuUagacccuaacuuguga......................................................................   4      1    Mm1
...................ucAcugagacccuaacuuguga......................................................................   3      1    Mm1
...................ucccugagacccuGacuuguga......................................................................   3      1    Mm1
...................ucccugCgacccuaacuuguga......................................................................   3      1    Mm1
...................ucccugagacccuaacuugugU......................................................................   292    1    Mm1
...................ucccugagacccuaGcuuguga......................................................................   4      1    Mm1
...................ucccugagacccuUacuuguga......................................................................   2      1    Mm1
...................ucccugagacccuaacAuguga......................................................................   26     1    Mm1
...................ucccugagacccuaacGuguga......................................................................   43     1    Mm1
...................Ncccugagacccuaacuuguga......................................................................   3      1    Mm1
...................Acccugagacccuaacuuguga......................................................................   2      1    Mm1
...................ucccugagaUccuaacuuguga......................................................................   26     1    Mm1
...................ucccugagacccuaacuGguga......................................................................   60     1    Mm1
...................ucccugagacGcuaacuuguga......................................................................   22     1    Mm1
...................ucccugagacccuaacuAguga......................................................................   75     1    Mm1
...................ucccugagacccuaacuugCga......................................................................   1      1    Mm1
...................uGccugagacccuaacuuguga......................................................................   1      1    Mm1
...................ucccAgagacccuaacuuguga......................................................................   1      1    Mm1
...................ucccugaAacccuaacuuguga......................................................................   2      1    Mm1
...................uccUugagacccuaacuuguga......................................................................   1      1    Mm1
...................ucccugagacccuaacuugugC......................................................................   4      1    Mm1
...................ucccugagacUcuaacuuguga......................................................................   67     1    Mm1
...................ucccugagGcccuaacuuguga......................................................................   3      1    Mm1
...................ucccugagacccuaacuuguAa......................................................................   2      1    Mm1
...................uAccugagacccuaacuuguga......................................................................   5      1    Mm1
...................ucccugagacccuaacCuguga......................................................................   31     1    Mm1
...................ucccugagacccuaacuCguga......................................................................   95     1    Mm1
...................ucccugaUacccuaacuuguga......................................................................   2      1    Mm1
...................ucccugagaGccuaacuuguga......................................................................   1      1    Mm1
...................ucGcugagacccuaacuuguga......................................................................   2      1    Mm1
...................ucccCgagacccuaacuuguga......................................................................   3      1    Mm1
...................ucccugagacccuaaUuuguga......................................................................   1      1    Mm1
...................ucccugagCcccuaacuuguga......................................................................   2      1    Mm1
...................ucccugagacccuaacuuUuga......................................................................   3      1    Mm1
...................ucccugagacccuaacuuguUa......................................................................   5      1    Mm1
...................ucccugagUcccuaacuuguga......................................................................   10     1    Mm1
...................ucccuNagacccuaacuuguga......................................................................   1      1    Mm1
...................ucccugagacAcuaacuuguga......................................................................   23     1    Mm1
...................ucccugagaccUuaacuuguga......................................................................   20     1    Mm1
...................ucccugagacccuaacuuguga......................................................................   14018  0    Mm1
...................ucccuAagacccuaacuuguga......................................................................   3      1    Mm1
...................ucUcugagacccuaacuuguga......................................................................   5      1    Mm1
...................ucccuCagacccuaacuuguga......................................................................   1      1    Mm1
...................ucccugagaAccuaacuuguga......................................................................   2      1    Mm1
...................ucccugGgacccuaacuuguga......................................................................   2      1    Mm1
...................ucccugagacccAaacuuguga......................................................................   2      1    Mm1
...................ucccugagacccuaacuugAga......................................................................   2      1    Mm1
...................ucccugagacccuaacuugugaA.....................................................................   691    1    Mm1
...................ucccugagacAcuaacuugugau.....................................................................   1      1    Mm1
...................ucccugagacccuaacuugugUu.....................................................................   6      1    Mm1
...................ucccugagacccuGacuugugau.....................................................................   1      1    Mm1
...................ucccugagacccuaacuugugau.....................................................................   164    0    Mm1
...................ucccugagacccuaacuugugaG.....................................................................   159    1    Mm1
...................ucccugagacccuaacuugugaAg....................................................................   4      1    Mm1
```

Mature Star

```
cguggaggcuuccuccccuucccugagacccuaacuugugauguaauaaaauucccggguuauggucuugggaauagggugguugcuauggcaacggaauacgacuaucacc
...................ucccugagacccuaacuugugauA.....................................................................    5      1    Mm1
...................ucccugagacccuaacuugugauU.....................................................................    35     1    Mm1
....................cccugagacccuaacuugugG.......................................................................    2      1    Mm1
....................cccugagacccuaacuuguga.......................................................................    18     0    Mm1
....................Uccugagacccuaacuuguga.......................................................................    1      1    Mm1
....................cccugagacccuaacuCguga.......................................................................    1      1    Mm1
......................cugagacccuaacuugugaG......................................................................    11     1    Mm1
......................................................ccggguuauggucuugggaaua....................................    9      0    Mm1

..................Cucccugagacccuaacuuguga.......................................................................    8      1    Mm2
...................ucccugagacccuaacuu...........................................................................    11     0    Mm2
...................ucccugagacGcuaacuu...........................................................................    1      1    Mm2
...................ucccugagacAcuaacuug..........................................................................    1      1    Mm2
...................ucccugagaAccuaacuug..........................................................................    1      1    Mm2
...................ucccugagacccuaacuug..........................................................................    60     0    Mm2
...................ucccugagacccuaacuuA..........................................................................    2      1    Mm2
...................ucccugagaUccuaacuugu.........................................................................    3      1    Mm2
...................ucccugagacccuaacuGgu.........................................................................    7      1    Mm2
...................ucccugagacccuaacuugG.........................................................................    8      1    Mm2
...................ucGcugagacccuaacuugu.........................................................................    1      1    Mm2
...................ucccugagacccuaacAugu.........................................................................    3      1    Mm2
...................ucccugagacccuaacGugu.........................................................................    5      1    Mm2
...................ucccugagacccuaacuugA.........................................................................    1      1    Mm2
...................ucccugagacccuaacuCgu.........................................................................    10     1    Mm2
...................ucccCgagacccuaacuugu.........................................................................    1      1    Mm2
...................ucccugagacccuUacuugu.........................................................................    1      1    Mm2
...................ucccugagacUcuaacuugu.........................................................................    9      1    Mm2
...................ucccugagacccuaaUuugu.........................................................................    2      1    Mm2
...................ucccugagacAcuaacuugu.........................................................................    2      1    Mm2
...................ucccugagacccuaacuugu.........................................................................    1361   0    Mm2
...................ucccugagacccuaacuAgu.........................................................................    5      1    Mm2
...................uccAugagacccuaacuugu.........................................................................    2      1    Mm2
...................ucccugagacccuaaAuugu.........................................................................    1      1    Mm2
...................ucccugagacGcuaacuugu.........................................................................    4      1    Mm2
...................ucccugagacccuaacCugu.........................................................................    6      1    Mm2
...................uAccugagacccuaacuugu.........................................................................    4      1    Mm2
...................ucccugagCcccuaacuugu.........................................................................    1      1    Mm2
...................ucccugaUacccuaacuugu.........................................................................    2      1    Mm2
...................ucccuAagacccuaacuugu.........................................................................    1      1    Mm2
...................ucccuUagacccuaacuugu.........................................................................    1      1    Mm2
...................ucccugaAacccuaacuugug........................................................................    2      1    Mm2
...................ucUcugagacccuaacuugug........................................................................    1      1    Mm2
...................Acccugagacccuaacuugug........................................................................    2      1    Mm2
...................ucccugagacUcuaacuugug........................................................................    11     1    Mm2
...................Ncccugagacccuaacuugug........................................................................    3      1    Mm2
...................ucccugagacccuaacuAgug........................................................................    13     1    Mm2
...................ucccugagaAccuaacuugug........................................................................    1      1    Mm2
...................ucccugagacccuaacuugug........................................................................    2159   0    Mm2
...................ucccugagaccGuaacuugug........................................................................    2      1    Mm2
...................ucccugagacccuaacAugug........................................................................    3      1    Mm2
...................uUccugagacccuaacuugug........................................................................    3      1    Mm2
...................ucccugagacccuaacuugGg........................................................................    2      1    Mm2
...................ucccugagacccuaacuuguC........................................................................    1      1    Mm2
...................ucccugagacGcuaacuugug........................................................................    7      1    Mm2
...................ucccugagacccuaacGugug........................................................................    10     1    Mm2
...................ucccugagacccuaacuuAug........................................................................    1      1    Mm2
...................Ccccugagacccuaacuugug........................................................................    2      1    Mm2
...................ucccugagacccuaacuGgug........................................................................    14     1    Mm2
...................ucGcugagacccuaacuugug........................................................................    1      1    Mm2
...................ucccugagacccuaacuugCg........................................................................    1      1    Mm2
...................ucccugagacccuaacCugug........................................................................    9      1    Mm2
...................ucccugagaccUuaacuugug........................................................................    3      1    Mm2
...................ucccugagacccuaacuCgug........................................................................    10     1    Mm2
...................ucccugUgacccuaacuugug........................................................................    1      1    Mm2
...................ucccugagaUccuaacuugug........................................................................    2      1    Mm2
...................ucccugagacccuaacuuguA.......................................................................    8      1    Mm2
...................ucccugagacAcuaacuugug........................................................................    4      1    Mm2
...................ucccugagUcccuaacuugug........................................................................    1      1    Mm2
...................ucccuUagacccuaacuugug........................................................................    3      1    Mm2
...................ucccugagacccuaacuuguU.......................................................................    18     1    Mm2
```

```
                   Mature             Star
cguggaggcuuccuccccuucccugagacccuaacuugugauguaauaaaauucccggguuauggucuugggaauagggugguugcuauggcaacggaauacgacuaucacc
...................ucccugagacccuaacuCguga.......................................................................  75     1   Mm2
...................ucccugagacccuaacuuUuga.......................................................................  1      1   Mm2
...................uccGugagacccuaacuuguga.......................................................................  2      1   Mm2
...................ucccugagacccuGacuuguga.......................................................................  4      1   Mm2
...................Ncccugagacccuaacuuguga.......................................................................  10     1   Mm2
...................ucccugagacccuaacuAguga.......................................................................  71     1   Mm2
...................ucccugagacccuaaUuuguga.......................................................................  5      1   Mm2
...................ucccugNgacccuaacuuguga.......................................................................  1      1   Mm2
...................Gcccugagacccuaacuuguga.......................................................................  1      1   Mm2
...................ucccugagaccAuaacuuguga.......................................................................  2      1   Mm2
...................ucccugaAacccuaacuuguga.......................................................................  2      1   Mm2
...................ucccugagacccuaacGuguga.......................................................................  34     1   Mm2
...................ucccuNagacccuaacuuguga.......................................................................  2      1   Mm2
...................ucccugagaUccuaacuuguga.......................................................................  18     1   Mm2
...................ucccCgagacccuaacuuguga.......................................................................  4      1   Mm2
...................uccAugagacccuaacuuguga.......................................................................  3      1   Mm2
...................ucccugagaAccuaacuuguga.......................................................................  2      1   Mm2
...................ucccugagacccuaacCuguga.......................................................................  37     1   Mm2
...................ucccugagacccuaacuuguUa.......................................................................  4      1   Mm2
...................ucccuUagacccuaacuuguga.......................................................................  1      1   Mm2
...................ucccugGgacccuaacuuguga.......................................................................  1      1   Mm2
...................ucccugagacccuaacuugugU.......................................................................  362    1   Mm2
...................uccUugagacccuaacuuguga.......................................................................  3      1   Mm2
...................ucccugagGcccuaacuuguga.......................................................................  1      1   Mm2
...................ucccuAagacccuaacuuguga.......................................................................  1      1   Mm2
...................ucccugagacccuaacuugCga.......................................................................  3      1   Mm2
...................Acccugagacccuaacuuguga.......................................................................  2      1   Mm2
...................ucccugagacccuaacuugGga.......................................................................  1      1   Mm2
...................ucccugagaccUuaacuuguga.......................................................................  6      1   Mm2
...................ucccugagacccuaacuuguAa.......................................................................  2      1   Mm2
...................ucccugagacccuaCcuuguga.......................................................................  1      1   Mm2
...................ucccugagCccuaacuuguga........................................................................  5      1   Mm2
...................ucccugagaGccuaacuuguga.......................................................................  1      1   Mm2
...................ucccugagacAcuaacuuguga.......................................................................  21     1   Mm2
...................ucccugaUacccuaacuuguga.......................................................................  1      1   Mm2
...................ucccugagacccuaacuugAga.......................................................................  1      1   Mm2
...................ucccugagacccuaacuugugC.......................................................................  8      1   Mm2
...................Ccccugagacccuaacuuguga.......................................................................  5      1   Mm2
...................ucccugagUccuaacuuguga........................................................................  10     1   Mm2
...................ucccugagacccuaacuugugG.......................................................................  4467   1   Mm2
...................ucccugagacccuaacAuguga.......................................................................  27     1   Mm2
...................ucGcugagacccuaacuuguga.......................................................................  7      1   Mm2
...................ucccugagacccuaacuuguga.......................................................................  14031  0   Mm2
...................ucccugUgacccuaacuuguga.......................................................................  3      1   Mm2
...................uUccugagacccuaacuuguga.......................................................................  5      1   Mm2
...................ucUcugagacccuaacuuguga.......................................................................  4      1   Mm2
...................ucccugagacGcuaacuuguga.......................................................................  19     1   Mm2
...................ucAcugagacccuaacuuguga.......................................................................  6      1   Mm2
...................ucccugagacUcuaacuuguga.......................................................................  140    1   Mm2
...................uAccugagacccuaacuuguga.......................................................................  9      1   Mm2
...................ucccugagacccuaacuGguga.......................................................................  50     1   Mm2
...................ucccugagacUcuaacuugugau......................................................................  3      1   Mm2
...................ucccugagacccuaacuugugUu......................................................................  3      1   Mm2
...................ucccugagacccuaacuugugaG......................................................................  380    1   Mm2
...................ucccugagacccuaacuGgugau......................................................................  1      1   Mm2
...................ucccugagacccuaacuugugau......................................................................  201    0   Mm2
...................ucccugagCcccuaacuugugau......................................................................  1      1   Mm2
...................ucccugagacAcuaacuugugau......................................................................  1      1   Mm2
...................ucccugagacccuaacuugugaA......................................................................  1401   1   Mm2
...................ucccugagacccuaacuCgugau......................................................................  2      1   Mm2
...................ucccugagacccuaacuuguUau......................................................................  1      1   Mm2
...................ucccugagacccuaacuugugaC......................................................................  3      1   Mm2
...................ucccugagacGcuaacuugugau......................................................................  1      1   Mm2
...................ucccugagGcccuaacuugugau......................................................................  1      1   Mm2
...................ucGcugagacccuaacuugugau......................................................................  1      1   Mm2
...................ucccugagacccuaacuugugaug.....................................................................  15     0   Mm2
...................ucccugagacccuaacuugugaAg.....................................................................  18     1   Mm2
...................ucccugagacccuaacuugugauA.....................................................................  2      1   Mm2
...................ucccugagacccuaacuugugauU.....................................................................  33     1   Mm2
...................ucccugagacccuaacuugugaugu....................................................................  1      0   Mm2
```

Mature **Star**

cguggaggcuuccuccccuucccugagacccuaacuugugauguaauaaaauucccggguuauggucuugggaauagggugguugcuauggcaacggaauacgacuaucacc			
...................ucccugagacccuaacuugugaugua...	4	0	Mm2
....................cccugagacccuaacuugu...	1	0	Mm2
....................cccugagacccuaacuuCug..	1	1	Mm2
....................cccugagacccuaacuugug..	2	0	Mm2
....................Uccugagacccuaacuuguga...	1	1	Mm2
....................cccCgagacccuaacuuguga...	1	1	Mm2
....................cccugagacccuaacuugugG...	12	1	Mm2
....................cccugagacccuaacuuguga...	24	0	Mm2
....................cccugagacccuaacuugugaA..	5	1	Mm2
....................cccugagacccuaacuugugau..	1	0	Mm2
.....................ccugagacccuaacuugug..	1	0	Mm2
.....................ccugagacccuaacuuguga...	17	0	Mm2
.....................ccugagacccuaacuugugG...	1	1	Mm2
.....................ccugagacUcuaacuuguga...	1	1	Mm2
.....................ccugagacccuaacuugugaG..	1	1	Mm2
.....................ccugagacccuaacuugugau..	1	0	Mm2
.......................ugagacccuaacuuguga...	6	0	Mm2
.......................ugagacccuaacuugugG...	1	1	Mm2
.......................ugagacccuaacuugugaG..	1	1	Mm2
.......................ugagacccuaacuugugaA..	2	1	Mm2
....uguaauaaaauucccggguuaugg...	1	0	Mm2
....ccggguuauggucuugggaaua....................................	22	0	Mm2
....ccggguuauggucuuggCaaua....................................	1	1	Mm2
....ccggguuauggucuuggUaaua....................................	1	1	Mm2
....ccggguuauggucuugggaaCa....................................	1	1	Mm2
....ccggguuauggucuugggaauaU...................................	2	1	Mm2
..................Cucccugagacccuaacuuguga...	9	1	Fm2
...................ucccugagacccuaacuA...	3	1	Fm2
...................ucccugagacccuaacuu...	3	0	Fm2
...................ucccugagacccuaacuug..	17	0	Fm2
...................ucccugagacccuaacuAg..	1	1	Fm2
...................ucccugagacccuaacuGgu...	4	1	Fm2
...................ucccugagacccuaacCugu...	1	1	Fm2
...................ucccugagacAcuaacuugu...	1	1	Fm2
...................ucccugagacccuGacuugu...	1	1	Fm2
...................ucccugagacccuaacuugu...	193	0	Fm2
...................ucccugagacccuaacuugG...	4	1	Fm2
...................ucccugagacccuaacuuUu...	1	1	Fm2
...................ucccugagacccuaacuCgu...	2	1	Fm2
...................ucccugagacGcuaacuugug..	1	1	Fm2
...................Ncccugagacccuaacuugug..	1	1	Fm2
...................ucccugagacccuaacCugug..	1	1	Fm2
...................ucccugagacccuaacuugCg..	1	1	Fm2
...................ucccugagacccuGacuugug..	1	1	Fm2
...................ucccugagacccuaacuAgug..	3	1	Fm2
...................ucccugagacccuaacuCgug..	4	1	Fm2
...................ucccugagacccuaacuGgug..	6	1	Fm2
...................ucccugaUacccuaacuugug..	1	1	Fm2
...................Ccccugagacccuaacuugug..	1	1	Fm2
...................ucccugagacccuaacuugug..	801	0	Fm2
...................uAccugagacccuaacuugug..	1	1	Fm2
...................ucccugagacUcuaacuugug..	1	1	Fm2
...................ucccugagacccuaacuuguU..	14	1	Fm2
...................ucccugagacccuaacAugug..	1	1	Fm2
...................Ccccugagacccuaacuuguga...	2	1	Fm2
...................ucccuUagacccuaacuuguga...	5	1	Fm2
...................uccUugagacccuaacuuguga...	2	1	Fm2
...................ucccuNagacccuaacuuguga...	2	1	Fm2
...................Ncccugagacccuaacuuguga...	8	1	Fm2
...................ucccugagacccuaacGuguga...	48	1	Fm2
...................ucccugaAacccuaacuuguga...	1	1	Fm2
...................ucccugagacccuaacuuUuga...	2	1	Fm2
...................ucccugagUcccuaacuuguga...	5	1	Fm2
...................ucccugagacccuaacCuguga...	50	1	Fm2
...................ucccugagaGccuaacuuguga...	1	1	Fm2
...................ucccugagacccuaacuugugG...	6483	1	Fm2
...................ucccugagacccuaacuuguCa...	1	1	Fm2
...................uUccugagacccuaacuuguga...	3	1	Fm2
...................ucccGgagacccuaacuuguga...	1	1	Fm2

```
                   Mature                             Star
cguggaggcuuccuccccuucccugagacccuaacuugugauguaauaaaauucccggguuauggucuugggaauagggugguugcuauggcaacggaauacgacuaucacc
...................ucccugagacUcuaacuuguga.......................................................................   65      1   Fm2
...................ucUcugagacccuaacuuguga.......................................................................   7       1   Fm2
...................ucccugagacccuCacuuguga.......................................................................   1       1   Fm2
...................ucccCgagacccuaacuuguga.......................................................................   7       1   Fm2
...................uccAugagacccuaacuuguga.......................................................................   7       1   Fm2
...................uAccugagacccuaacuuguga.......................................................................   8       1   Fm2
...................ucccugagacccuaacuAguga.......................................................................   102     1   Fm2
...................uGccugagacccuaacuuguga.......................................................................   3       1   Fm2
...................ucccugagacccuaacuuguAa.......................................................................   1       1   Fm2
...................ucccugagaccUuaacuuguga.......................................................................   3       1   Fm2
...................ucccugagacccuaacuugugU.......................................................................   104     1   Fm2
...................ucccugNgacccuaacuuguga.......................................................................   1       1   Fm2
...................ucccugagacccuaacuCguga.......................................................................   107     1   Fm2
...................ucccugagacccuGacuuguga.......................................................................   7       1   Fm2
...................ucccugagacccuaacuugGga.......................................................................   1       1   Fm2
...................ucccugagacccuaacuugAga.......................................................................   1       1   Fm2
...................ucccugagacccuaacAuguga.......................................................................   37      1   Fm2
...................ucccugagacccuaacuuguga.......................................................................   17480   0   Fm2
...................ucccugagacccuaacuuAuga.......................................................................   3       1   Fm2
...................ucccugagacAcuaacuuguga.......................................................................   38      1   Fm2
...................ucccugagacccuaGcuuguga.......................................................................   3       1   Fm2
...................ucccugagacccuaacuGguga.......................................................................   72      1   Fm2
...................ucccugagacccuaacuugugC.......................................................................   3       1   Fm2
...................ucccuCagacccuaacuuguga.......................................................................   1       1   Fm2
...................ucccugagaUccuaacuuguga.......................................................................   2       1   Fm2
...................ucccugagacccuaaGuuguga.......................................................................   1       1   Fm2
...................ucGcugagacccuaacuuguga.......................................................................   7       1   Fm2
...................ucccugagCcccuaacuuguga.......................................................................   3       1   Fm2
...................ucccugGgacccuaacuuguga.......................................................................   3       1   Fm2
...................ucccugagacGcuaacuuguga.......................................................................   28      1   Fm2
...................ucccuAagacccuaacuuguga.......................................................................   3       1   Fm2
...................ucccugagaccAuaacuuguga.......................................................................   4       1   Fm2
...................ucAcugagacccuaacuuguga.......................................................................   9       1   Fm2
...................ucccugagGcccuaacuuguga.......................................................................   4       1   Fm2
...................ucccugagaccGuaacuuguga.......................................................................   1       1   Fm2
...................ucccugaUacccuaacuuguga.......................................................................   5       1   Fm2
...................ucccugUgacccuaacuuguga.......................................................................   4       1   Fm2
...................ucccugagaAccuaacuuguga.......................................................................   2       1   Fm2
...................ucccugagacccCaacuuguga.......................................................................   3       1   Fm2
...................ucccugCgacccuaacuuguga.......................................................................   2       1   Fm2
...................ucccugagacccuaacuuguUa.......................................................................   9       1   Fm2
...................ucccugagacccuaacuuCuga.......................................................................   1       1   Fm2
...................uNccugagacccuaacuuguga.......................................................................   3       1   Fm2
...................ucccugagacccuaacuugugaG......................................................................   546     1   Fm2
...................ucccugagacccuaacuAgugau......................................................................   1       1   Fm2
...................ucccugagacAcuaacuugugau......................................................................   3       1   Fm2
...................ucccugagacccuaacuugugau......................................................................   83      0   Fm2
...................ucccugagacccuaacuugugUu......................................................................   8       1   Fm2
...................ucccugagacccuaacuugugaA......................................................................   630     1   Fm2
...................ucccugagacccuaacGugugau......................................................................   1       1   Fm2
...................ucUcugagacccuaacuugugau......................................................................   1       1   Fm2
....................cccugagacccuaacuugug........................................................................   10      0   Fm2
....................cccugagacccuaacuugugG.......................................................................   3       1   Fm2
....................cccugagacccuaacuuguga.......................................................................   5       0   Fm2
....................cccugagacccuaacuugugaA......................................................................   6       1   Fm2
.....................ccugagacccuaacuuguga.......................................................................   8       0   Fm2
.....................ccugagacccuaacuugugG.......................................................................   1       1   Fm2
......................................................ccggguuauggucuugggaaua....................................   39      0   Fm2
..........................................................guuauggucuugggaaua....................................   6       0   Fm2

...................ucccugagacccuaacuu...........................................................................   18      0   Fm1
...................ucccugagacAcuaacuu...........................................................................   1       1   Fm1
...................ucccugagacccuaacuug..........................................................................   32      0   Fm1
...................ucccugagacccuaacuAg..........................................................................   1       1   Fm1
...................ucccugagacccuaacuAgu.........................................................................   4       1   Fm1
...................ucccugagacccuaacuCgu.........................................................................   4       1   Fm1
...................ucccugagacccuGacuugu.........................................................................   1       1   Fm1
...................ucccugagUcccuaacuugu.........................................................................   1       1   Fm1
...................ucccugagacUcuaacuugu.........................................................................   3       1   Fm1
...................ucccugagacGcuaacuugu.........................................................................   3       1   Fm1
```

Mature **Star**

cguggaggcuuccuccccuucccugagacccuaacuugugauguaauaaauucccggguuauggucuugggaauagggugguugcuauggcaacggaauacgacuaucacc			
...................ucccugagacccuaacCugu..	4	1	Fm1
...................ucccugagacAcuaacuugu..	1	1	Fm1
...................ucccugagacccuaacGugu..	1	1	Fm1
...................uccAugagacccuaacuugu..	1	1	Fm1
...................ucccugagaUccuaacuugu..	3	1	Fm1
...................ucccugagaccUuaacuugu..	3	1	Fm1
...................ucccugagacccuaacuugG..	3	1	Fm1
...................ucccugagacccuaacuugu..	738	0	Fm1
...................ucccugagacccuaacuGgu..	2	1	Fm1
...................ucccugagacccuaacuuguA...	1	1	Fm1
...................ucccugagacAcuaacuugug...	1	1	Fm1
...................ucccugagacccuaacuAgug...	2	1	Fm1
...................ucccugagacUcuaacuugug...	13	1	Fm1
...................uAccugagacccuaacuugug...	1	1	Fm1
...................ucccugagaUccuaacuugug...	3	1	Fm1
...................ucccugagaccUuaacuugug...	1	1	Fm1
...................ucccugagacccuaacAugug...	3	1	Fm1
...................ucGcugagacccuaacuugug...	1	1	Fm1
...................ucccugagacccuaacCugug...	2	1	Fm1
...................ucccugagaGccuaacuugug...	1	1	Fm1
...................ucccugagacccuaacuugug...	918	0	Fm1
...................ucccugagacccuaacGugug...	2	1	Fm1
...................ucccugagacccuaacuuguU...	11	1	Fm1
...................uUccugagacccuaacuugug...	1	1	Fm1
...................ucAcugagacccuaacuugug...	1	1	Fm1
...................ucUcugagacccuaacuugug...	1	1	Fm1
...................ucccuNagacccuaacuugug...	1	1	Fm1
...................ucccugaUacccuaacuugug...	1	1	Fm1
...................ucccugagacccuaacuGgug...	4	1	Fm1
...................ucccugagacccuaacuCgug...	6	1	Fm1
...................ucccugagacccuaacuCguga..	122	1	Fm1
...................ucccuAagacccuaacuuguga..	10	1	Fm1
...................ucccugNgacccuaacuuguga..	1	1	Fm1
...................ucccugagacccuCacuuguga..	2	1	Fm1
...................ucccugaCacccuaacuuguga..	1	1	Fm1
...................ucGcugagacccuaacuuguga..	10	1	Fm1
...................ucccugagacccuaacuugugC..	8	1	Fm1
...................ucccugagacccuaacGuguga..	73	1	Fm1
...................ucccugagacccuaacuAguga..	104	1	Fm1
...................ucccCgagacccuaacuuguga..	9	1	Fm1
...................ucccugagCcccuaacuuguga..	2	1	Fm1
...................ucccugagacccuaacuuUuga..	5	1	Fm1
...................ucccugagacAcuaacuuguga..	42	1	Fm1
...................ucUcugagacccuaacuuguga..	5	1	Fm1
...................ucccugagacccuaacuugugU..	150	1	Fm1
...................ucccugagacccuaacuuguga..	23074	0	Fm1
...................ucccugaAacccuaacuuguga..	2	1	Fm1
...................ucccugagaUccuaacuuguga..	14	1	Fm1
...................ucccugagacccGaacuuguga..	2	1	Fm1
...................uGccugagacccuaacuuguga..	1	1	Fm1
...................ucccugagacccuaacuuAuga..	1	1	Fm1
...................ucccugGgacccuaacuuguga..	2	1	Fm1
...................ucccugCgacccuaacuuguga..	3	1	Fm1
...................ucccAgagacccuaacuuguga..	1	1	Fm1
...................ucccGgagacccuaacuuguga..	1	1	Fm1
...................ucccugagacccuaacuuguUa..	1	1	Fm1
...................ucccugagaccUuaacuuguga..	8	1	Fm1
...................ucccugagacccuaacuugugG..	11236	1	Fm1
...................uAccugagacccuaacuuguga..	6	1	Fm1
...................Acccugagacccuaacuuguga..	3	1	Fm1
...................ucccugagacccuaaUuuguga..	5	1	Fm1
...................ucccugagacccuaUcuuguga..	1	1	Fm1
...................ucccuNagacccuaacuuguga..	2	1	Fm1
...................ucccugagacccuaacuGguga..	92	1	Fm1
...................ucccugagacccCaacuuguga..	3	1	Fm1
...................uccUugagacccuaacuuguga..	6	1	Fm1
...................uccAugagacccuaacuuguga..	7	1	Fm1
...................ucccuCagacccuaacuuguga..	1	1	Fm1
...................ucccugagacccuaacuuguCa..	2	1	Fm1
...................ucccugagacccuaacCuguga..	75	1	Fm1

Mature Star

cguggaggcuuccuccccuucccugagacccuaacuugugauguaauaaaauucccggguuauggucuugggaauagggugguugcuauggcaacggaauacgacuaucacc			
...................ucccugagacccuaacuuguAa...	5	1	Fm1
...................ucccugagaccGuaacuuguga...	1	1	Fm1
...................ucAcugagacccuaacuuguga...	8	1	Fm1
...................uUccugagacccuaacuuguga...	4	1	Fm1
...................ucccugagUcccuaacuuguga...	13	1	Fm1
...................ucccuUagacccuaacuuguga...	6	1	Fm1
...................ucccugagacGcuaacuuguga...	25	1	Fm1
...................ucccugagaGccuaacuuguga...	3	1	Fm1
...................ucccugaUacccuaacuuguga...	1	1	Fm1
...................ucccugagacccuGacuuguga...	6	1	Fm1
...................ucccugagaAccuaacuuguga...	2	1	Fm1
...................Ncccugagacccuaacuuguga...	9	1	Fm1
...................ucccugagacccuaacuugCga...	1	1	Fm1
...................ucccugagGcccuaacuuguga...	4	1	Fm1
...................ucccugagacccuaacuugAga...	2	1	Fm1
...................ucccugagacccuaacAuguga...	59	1	Fm1
...................ucccugagacccuaGcuuguga...	1	1	Fm1
...................Ccccugagacccuaacuuguga...	5	1	Fm1
...................ucccugUgacccuaacuuguga...	5	1	Fm1
...................ucccugagacccuUacuuguga...	2	1	Fm1
...................ucccugagacUcuaacuuguga...	81	1	Fm1
...................ucccugagacccuaacuuCuga...	2	1	Fm1
...................ucccugagaccAuaacuuguga...	4	1	Fm1
...................ucccugagacUcuaacuugugau..	6	1	Fm1
...................ucccugagacccuaacuugugaG..	167	1	Fm1
...................ucUcugagacccuaacuugugau..	3	1	Fm1
...................ucccugagacccuaacuGgugau..	1	1	Fm1
...................ucccugagaccUuaacuugugau..	2	1	Fm1
...................ucccugagacccuaacuugugUu..	6	1	Fm1
...................ucccugagacccuaacuugugaC..	4	1	Fm1
...................ucccugagacccuaacuugugau..	79	0	Fm1
...................ucccugagacccuaacuugugaA..	615	1	Fm1
...................ucccugagacccuaacuugugGu..	1	1	Fm1
...................ucccugagacccuaacuugugauU...	13	1	Fm1
...................ucccugagacccuaacuugugaAg...	7	1	Fm1
...................ucccugagacccuaacuugugaugu..	5	0	Fm1
...................ucccugagacccuaacuugugauguaauaaaauuc..	1	0	Fm1
....................cccugagacccuaacuuCug..	1	1	Fm1
....................cccugagacccuaacuugug..	7	0	Fm1
....................cccugagacccuaacuugugG...	3	1	Fm1
....................Uccugagacccuaacuuguga...	1	1	Fm1
.....................ccugagacccuaacuugu...	1	0	Fm1
.....................ccugagacccuaacAugu...	1	1	Fm1
.....................ccugagacccuaacuuguU..	5	1	Fm1
.....................ccugagacccuaacuugugG...	4	1	Fm1
.....................ccugagacccuaacuuguga...	3	0	Fm1
.....................ccugagacccuaacuugugaA..	1	1	Fm1
.......................ugagacccuaacuugugG...	3	1	Fm1
.......................ugagacccuaacuuguga...	6	0	Fm1
.......................ugagacccuaacuugugaG..	2	1	Fm1
....ccggguuauggucuugggaa......................................	15	0	Fm1
....ccUgguuauggucuugggaau.....................................	1	1	Fm1
....ccggguuauggucuugCgaau.....................................	1	1	Fm1
....ccggguuauggucuugggaau.....................................	19	0	Fm1
....ccggguuauggucuugggaauU....................................	7	1	Fm1
....ccggguuauggucuugggaaua....................................	47	0	Fm1
....ccggguuauggucuuggAaaua....................................	1	1	Fm1
....ccggguuauggucuuggCaaua....................................	1	1	Fm1
....gguuauggucuugggaaua....................................	1	0	Fm1
..................Cucccugagacccuaacuugu...	1	1	Mm3
..................uucccugagacccuaacuugu...	1	0	Mm3
..................Cucccugagacccuaacuuguga...	4	1	Mm3
...................ucccugagacccuaacuu...	27	0	Mm3
...................ucccugagacccuaacuA...	1	1	Mm3
...................ucccugagacccuaacuGg..	1	1	Mm3
...................ucccugagacccuaacuCg..	1	1	Mm3
...................ucccugagacccuaacuAg..	2	1	Mm3
...................ucccugagacGcuaacuug..	1	1	Mm3
...................ucccugagacccuaacCug..	2	1	Mm3

Mature Star

cguggaggcuuccuccccuucccugagacccuaacuugugauguaauaaauucccggguuauggucuugggaauagggugguugcuauggcaacggaauacgacuaucacc			
...................ucccugagacccuaacuug...	112	0	Mm3
...................ucccugagacccuaacuuU...	4	1	Mm3
...................ucccugagaUccuaacuug...	2	1	Mm3
...................ucccugagacccuaacuuA...	4	1	Mm3
...................ucccugagaUccuaacuugu..	4	1	Mm3
...................ucccugagacccCaacuugu..	1	1	Mm3
...................uccAugagacccuaacuugu..	1	1	Mm3
...................ucccugagaccUuaacuugu..	5	1	Mm3
...................ucAcugagacccuaacuugu..	2	1	Mm3
...................ucccugagacccuaacuCgu..	8	1	Mm3
...................ucccugagacccuaacuugu..	1793	0	Mm3
...................ucccugagacccuaacuugC..	1	1	Mm3
...................ucccugagacUcuaacuugu..	8	1	Mm3
...................ucUcugagacccuaacuugu..	2	1	Mm3
...................ucccugagacccuaacCugu..	4	1	Mm3
...................ucccugagCcccuaacuugu..	1	1	Mm3
...................ucGcugagacccuaacuugu..	2	1	Mm3
...................ucccugagacccuaacuAgu..	4	1	Mm3
...................ucccugagacGcuaacuugu..	1	1	Mm3
...................uAccugagacccuaacuugu..	1	1	Mm3
...................ucccugagacccuaacAugu..	6	1	Mm3
...................ucccugagacccuaacuugG..	9	1	Mm3
...................Ncccugagacccuaacuugu..	1	1	Mm3
...................ucccugagGcccuaacuugu..	1	1	Mm3
...................ucccugUgacccuaacuugu..	1	1	Mm3
...................ucccugagacAcuaacuugu..	5	1	Mm3
...................ucccugagacccuaacGugu..	4	1	Mm3
...................ucccugagacccuaacuGgu..	5	1	Mm3
...................ucccugagacccuaacuuCu..	1	1	Mm3
...................uUccugagacccuaacuugu..	2	1	Mm3
...................ucccugagacccuGacuugu..	1	1	Mm3
...................ucccugagacccGaacuugu..	1	1	Mm3
...................ucccuUagacccuaacuugu..	1	1	Mm3
...................Acccugagacccuaacuugug...	1	1	Mm3
...................ucccugagacAcuaacuugug...	4	1	Mm3
...................ucccugagacUcuaacuugug...	12	1	Mm3
...................ucccugagUcccuaacuugug...	1	1	Mm3
...................ucccugagacccuaacuAgug...	22	1	Mm3
...................ucccugagacccuaacCugug...	16	1	Mm3
...................ucccugagacccuaacuGgug...	28	1	Mm3
...................ucccugUgacccuaacuugug...	1	1	Mm3
...................ucccugagCcccuaacuugug...	1	1	Mm3
...................ucccugagacccuaacuugGg...	2	1	Mm3
...................ucccugagaUccuaacuugug...	4	1	Mm3
...................Ccccugagacccuaacuugug...	2	1	Mm3
...................ucccugNgacccuaacuugug...	2	1	Mm3
...................ucccugagacccuaacuuguU...	28	1	Mm3
...................ucGcugagacccuaacuugug...	1	1	Mm3
...................ucccugagaAccuaacuugug...	2	1	Mm3
...................ucccugagacccuaacuuguC...	1	1	Mm3
...................ucccugagacccuaacuugCg...	1	1	Mm3
...................ucccugagacccuaaAuugug...	1	1	Mm3
...................ucccugagacccAaacuugug...	1	1	Mm3
...................uUccugagacccuaacuugug...	2	1	Mm3
...................uAccugagacccuaacuugug...	2	1	Mm3
...................ucccugagaccUuaacuugug...	2	1	Mm3
...................Ncccugagacccuaacuugug...	1	1	Mm3
...................ucccugagacccuaacuuguA...	13	1	Mm3
...................ucccugagacGcuaacuugug...	3	1	Mm3
...................ucccugagacccuaacuuAug...	2	1	Mm3
...................ucAcugagacccuaacuugug...	1	1	Mm3
...................ucccugaAacccuaacuugug...	2	1	Mm3
...................ucccugagacccuaacGugug...	11	1	Mm3
...................ucccugagacccuaacuCgug...	28	1	Mm3
...................ucccugagacccuaacuuUug...	1	1	Mm3
...................ucccugagacccuaaUuugug...	1	1	Mm3
...................uGccugagacccuaacuugug...	1	1	Mm3
...................ucccugagacccuaGcuugug...	1	1	Mm3
...................ucUcugagacccuaacuugug...	1	1	Mm3
...................ucccugagacccuaacAugug...	7	1	Mm3

```
                   Mature                              Star
cguggaggcuuccuccccuucccugagacccuaacuugugauguaauaaauucccggguuauggucuugggaauagggugguugcuauggcaacggaauacgacuaucacc
...................ucccCgagacccuaacuugug.......................................................................   2      1    Mm3
...................uccUugagacccuaacuugug.......................................................................   1      1    Mm3
...................ucccugagacccuaacuugug.......................................................................   4743   0    Mm3
...................ucccugUgacccuaacuuguga......................................................................   11     1    Mm3
...................ucccugagacccuaacuugugG......................................................................   4700   1    Mm3
...................ucccuAagacccuaacuuguga......................................................................   1      1    Mm3
...................uGccugagacccuaacuuguga......................................................................   3      1    Mm3
...................ucccugagacccuaacuuguUa......................................................................   2      1    Mm3
...................ucccugagaccGuaacuuguga......................................................................   2      1    Mm3
...................ucccugagaGccuaacuuguga......................................................................   1      1    Mm3
...................ucccugagGcccuaacuuguga......................................................................   4      1    Mm3
...................ucccugagacccuaUcuuguga......................................................................   4      1    Mm3
...................ucccugagacccuaaUuuguga......................................................................   1      1    Mm3
...................Ncccugagacccuaacuuguga......................................................................   16     1    Mm3
...................ucccugagacccuaacuugAga......................................................................   2      1    Mm3
...................ucccugagacccCaacuuguga......................................................................   4      1    Mm3
...................ucccugagacccuaacuuAuga......................................................................   3      1    Mm3
...................ucAcugagacccuaacuuguga......................................................................   7      1    Mm3
...................ucccugagacccuaacuuguga......................................................................   26241  0    Mm3
...................ucccugCgacccuaacuuguga......................................................................   4      1    Mm3
...................ucccugagacccuaacuugCga......................................................................   4      1    Mm3
...................ucccugagacGcuaacuuguga......................................................................   31     1    Mm3
...................ucUcugagacccuaacuuguga......................................................................   8      1    Mm3
...................ucccugaCacccuaacuuguga......................................................................   1      1    Mm3
...................ucccugagacccuaacuugGga......................................................................   1      1    Mm3
...................ucccAgagacccuaacuuguga......................................................................   3      1    Mm3
...................uAccugagacccuaacuuguga......................................................................   15     1    Mm3
...................ucccugagaUccuaacuuguga......................................................................   17     1    Mm3
...................ucccugagacccAaacuuguga......................................................................   1      1    Mm3
...................ucccugagacccuaacuCguga......................................................................   171    1    Mm3
...................Ccccugagacccuaacuuguga......................................................................   2      1    Mm3
...................ucccuCagacccuaacuuguga......................................................................   1      1    Mm3
...................ucccugagacccuaacuugugC......................................................................   9      1    Mm3
...................ucccugagCcccuaacuuguga......................................................................   5      1    Mm3
...................ucccugagacccuaacuuguCa......................................................................   2      1    Mm3
...................ucccugagacAcuaacuuguga......................................................................   47     1    Mm3
...................ucccugagacccuaacGuguga......................................................................   90     1    Mm3
...................ucccugaUacccuaacuuguga......................................................................   6      1    Mm3
...................ucccugagacUcuaacuuguga......................................................................   622    1    Mm3
...................ucGcugagacccuaacuuguga......................................................................   14     1    Mm3
...................ucccugagacccuUacuuguga......................................................................   1      1    Mm3
...................ucccugagacccuaacuuUuga......................................................................   5      1    Mm3
...................ucccugagacccuGacuuguga......................................................................   3      1    Mm3
...................ucccugagacccuaacuAguga......................................................................   139    1    Mm3
...................ucccuNagacccuaacuuguga......................................................................   6      1    Mm3
...................ucccugaAacccuaacuuguga......................................................................   2      1    Mm3
...................ucccugagacccuaacAuguga......................................................................   58     1    Mm3
...................uccAugagacccuaacuuguga......................................................................   8      1    Mm3
...................uNccugagacccuaacuuguga......................................................................   1      1    Mm3
...................ucccugagacccuaacuuCuga......................................................................   1      1    Mm3
...................ucccugagUcccuaacuuguga......................................................................   14     1    Mm3
...................ucccugGgacccuaacuuguga......................................................................   5      1    Mm3
...................ucccugagaccUuaacuuguga......................................................................   22     1    Mm3
...................ucccugagacccuaacCuguga......................................................................   78     1    Mm3
...................ucccGgagacccuaacuuguga......................................................................   2      1    Mm3
...................ucccugagacccuaacuugugU......................................................................   563    1    Mm3
...................ucccugagaAccuaacuuguga......................................................................   4      1    Mm3
...................ucccuUagacccuaacuuguga......................................................................   2      1    Mm3
...................ucccugagacccuaGcuuguga......................................................................   1      1    Mm3
...................ucccugagacccuaacuuguAa......................................................................   2      1    Mm3
...................ucccugagacccuaacuGguga......................................................................   125    1    Mm3
...................ucccCgagacccuaacuuguga......................................................................   6      1    Mm3
...................uUccugagacccuaacuuguga......................................................................   7      1    Mm3
...................ucccugagacccuaacuugugaC.....................................................................   3      1    Mm3
...................ucccugagacccuaacuGgugau.....................................................................   2      1    Mm3
...................ucccugagacccuaacCugugau.....................................................................   1      1    Mm3
...................ucccugagacccuaacuugugau.....................................................................   398    0    Mm3
...................ucccugagacccuaacuAgugau.....................................................................   1      1    Mm3
...................ucccugagacccuaacuugugUu.....................................................................   8      1    Mm3
...................ucccCgagacccuaacuugugau.....................................................................   1      1    Mm3
```

Mature **Star**

```
cguggaggcuuccuccccuucccugagacccuaacuugugauguaauaaauucccggguuauggucuugggaauagggugguugcuauggcaacggaauacgacuaucacc
...................ucccugagacccuaacAugugau.....................................................................   1      1    Mm3
...................ucccugagacccuaacuugugaA.....................................................................   3156   1    Mm3
...................ucccugagacUcuaacuugugau.....................................................................   2      1    Mm3
...................ucccugagacccuaacuCgugau.....................................................................   1      1    Mm3
...................ucUcugagacccuaacuugugau.....................................................................   1      1    Mm3
...................ucccugagacAcuaacuugugau.....................................................................   1      1    Mm3
...................ucccugagacccuaacuugugaG.....................................................................   299    1    Mm3
...................ucccugagacccuaacuugugaAg....................................................................   16     1    Mm3
...................ucccugagacccuaacuugugauA....................................................................   1      1    Mm3
...................ucccugagacccuaacuCgugaug....................................................................   1      1    Mm3
...................ucccugagacccuaacuugugaug....................................................................   21     0    Mm3
...................ucccugagacccuaacuugugauU....................................................................   47     1    Mm3
...................ucccugagacccuaacuugugaGg....................................................................   1      1    Mm3
...................ucccugagacccuaacuugugaugu...................................................................   2      0    Mm3
...................ucccugagacccuaacuugugauAu...................................................................   1      1    Mm3
...................ucccugagacccuaacuugugauguaauaaauuc..........................................................   1      0    Mm3
....................cccugagacccuaacuugu........................................................................   1      0    Mm3
....................cccugagacccuaacuugug.......................................................................   2      0    Mm3
....................cccugagacccuaacuuguga......................................................................   41     0    Mm3
....................Uccugagacccuaacuuguga......................................................................   1      1    Mm3
....................cccugagacUcuaacuuguga......................................................................   5      1    Mm3
....................cccugagacccuaacuuCuga......................................................................   1      1    Mm3
....................cccugagacccuaacuugugG......................................................................   5      1    Mm3
....................cccugagacccuaacuuAuga......................................................................   1      1    Mm3
....................cccugagacccuaacuugugaG.....................................................................   1      1    Mm3
....................cccugagacccuaacuugugaA.....................................................................   5      1    Mm3
.....................ccugagacccuaacuugu........................................................................   2      0    Mm3
.....................ccugagacccuaacuugug.......................................................................   5      0    Mm3
.....................ccugagacccuaaAuuguga......................................................................   1      1    Mm3
.....................ccugagacccuaacuuguga......................................................................   19     0    Mm3
.....................ccugagacccuaacuuguUa......................................................................   1      1    Mm3
.....................ccugagacccuaacuugugG......................................................................   3      1    Mm3
.....................ccugagacUcuaacuuguga......................................................................   2      1    Mm3
.....................ccugagacccuaacuugugaA.....................................................................   3      1    Mm3
......................cugagacccuaacuugug.......................................................................   1      0    Mm3
......................cugagacccuaacuuguga......................................................................   1      0    Mm3
.......................ugagacccuaacuuguga......................................................................   11     0    Mm3
.......................ugagacccuaacuugugG......................................................................   2      1    Mm3
.......................ugagacUcuaacuuguga......................................................................   1      1    Mm3
.......................ugagacccuaacuugugaG.....................................................................   2      1    Mm3
.......................ugagacccuaacuugugau.....................................................................   1      0    Mm3
.....................................................ccggguuauggucuuggg........................................   1      0    Mm3
.....................................................ccggguuauggucuugggaa......................................   1      0    Mm3
.....................................................ccggguuauggucuugggaau.....................................   2      0    Mm3
.....................................................ccggguuauggucuugggaaua....................................   5      0    Mm3

..................Cucccugagacccuaacuugu........................................................................   4      1    Fm3
..................Cucccugagacccuaacuuguga......................................................................   6      1    Fm3
...................ucccugagacccuaacAu..........................................................................   2      1    Fm3
...................ucccugagacccuaacuu..........................................................................   25     0    Fm3
...................ucGcugagacccuaacuu..........................................................................   1      1    Fm3
...................ucccugagacccuaacuA..........................................................................   1      1    Fm3
...................ucccugagacUcuaacuug.........................................................................   1      1    Fm3
...................ucAcugagacccuaacuug.........................................................................   1      1    Fm3
...................ucccugagacccuaacAug.........................................................................   1      1    Fm3
...................ucccugagacccuaacuug.........................................................................   472    0    Fm3
...................ucccugagacccuaacuGg.........................................................................   1      1    Fm3
...................ucccugagGcccuaacuug.........................................................................   1      1    Fm3
...................uAccugagacccuaacuug.........................................................................   1      1    Fm3
...................ucccugagacccuaacuuA.........................................................................   11     1    Fm3
...................ucccugagaUccuaacuug.........................................................................   6      1    Fm3
...................ucccugagacccuaacCug.........................................................................   2      1    Fm3
...................ucccugagacccuaacuuU.........................................................................   3      1    Fm3
...................ucccCgagacccuaacuug.........................................................................   1      1    Fm3
...................uccUugagacccuaacuugu........................................................................   2      1    Fm3
...................ucGcugagacccuaacuugu........................................................................   1      1    Fm3
...................ucccugagacccuaacuGgu........................................................................   35     1    Fm3
...................ucccugagacccuGacuugu........................................................................   1      1    Fm3
...................ucAcugagacccuaacuugu........................................................................   3      1    Fm3
...................ucccugagaGccuaacuugu........................................................................   1      1    Fm3
```

```
                  Mature                Star
cguggaggcuuccuccccuucccugagacccuaacuugugauguaauaaaauucccggguuauggucuugggaauagggugguugcuauggcaacggaauacgacuaucacc
...................ucccugagacccuaacuAgu.........................................................................  33    1  Fm3
...................ucccugagacccuaacAugu.........................................................................  16    1  Fm3
...................uGccugagacccuaacuugu.........................................................................  1     1  Fm3
...................ucccugGgacccuaacuugu.........................................................................  1     1  Fm3
...................Ncccugagacccuaacuugu.........................................................................  2     1  Fm3
...................uccAugagacccuaacuugu.........................................................................  4     1  Fm3
...................uUccugagacccuaacuugu.........................................................................  2     1  Fm3
...................ucccugagaAccuaacuugu.........................................................................  2     1  Fm3
...................ucccugagacccCaacuugu.........................................................................  1     1  Fm3
...................ucccuAagacccuaacuugu.........................................................................  1     1  Fm3
...................ucccuNagacccuaacuugu.........................................................................  1     1  Fm3
...................ucccugagacAcuaacuugu.........................................................................  12    1  Fm3
...................ucccugagacccuaacGugu.........................................................................  10    1  Fm3
...................ucccugagacccuaacuCgu.........................................................................  34    1  Fm3
...................ucccugagacccuaacuugu.........................................................................  5463  0  Fm3
...................ucccugagaccAuaacuugu.........................................................................  2     1  Fm3
...................ucccuUagacccuaacuugu.........................................................................  3     1  Fm3
...................ucccugCgacccuaacuugu.........................................................................  1     1  Fm3
...................ucccugagacUcuaacuugu.........................................................................  20    1  Fm3
...................ucccugagaUccuaacuugu.........................................................................  17    1  Fm3
...................ucccugagacccuaacuugA.........................................................................  4     1  Fm3
...................ucccugagacGcuaacuugu.........................................................................  5     1  Fm3
...................ucccugagaccUuaacuugu.........................................................................  4     1  Fm3
...................ucccugagacccAaacuugu.........................................................................  1     1  Fm3
...................ucccCgagacccuaacuugu.........................................................................  3     1  Fm3
...................ucccugagacccuUacuugu.........................................................................  1     1  Fm3
...................ucccugagacccuaGcuugu.........................................................................  1     1  Fm3
...................ucccAgagacccuaacuugu.........................................................................  2     1  Fm3
...................ucccugaAacccuaacuugu.........................................................................  1     1  Fm3
...................ucccugagacccuaacuuUu.........................................................................  2     1  Fm3
...................ucccugagacccuaacCugu.........................................................................  14    1  Fm3
...................ucccugagUccuaacuugug........................................................................  6     1  Fm3
...................ucccugUgacccuaacuugug........................................................................  2     1  Fm3
...................ucccugagacccuaacGugug........................................................................  16    1  Fm3
...................ucccugagaccAuaacuugug........................................................................  2     1  Fm3
...................ucccugagacccuaacuAgug........................................................................  43    1  Fm3
...................ucccugaAacccuaacuugug........................................................................  3     1  Fm3
...................ucccugagacccuaacuGgug........................................................................  27    1  Fm3
...................ucccugagacccuaacuugCg........................................................................  1     1  Fm3
...................ucccugagacccuaacuCgug........................................................................  37    1  Fm3
...................ucGcugagacccuaacuugug........................................................................  3     1  Fm3
...................ucccugaCacccuaacuugug........................................................................  1     1  Fm3
...................ucccugagacccuGacuugug........................................................................  1     1  Fm3
...................ucccugagacGcuaacuugug........................................................................  8     1  Fm3
...................uGccugagacccuaacuugug........................................................................  1     1  Fm3
...................ucccugagacUcuaacuugug........................................................................  13    1  Fm3
...................ucccugagacccuaacAugug........................................................................  13    1  Fm3
...................ucccugagacccCaacuugug........................................................................  2     1  Fm3
...................ucccugagacccuaacuugug........................................................................  6040  0  Fm3
...................uUccugagacccuaacuugug........................................................................  6     1  Fm3
...................ucccAgagacccuaacuugug........................................................................  1     1  Fm3
...................ucccugagacccuaacuuguA........................................................................  8     1  Fm3
...................uccUugagacccuaacuugug........................................................................  1     1  Fm3
...................ucccuUagacccuaacuugug........................................................................  1     1  Fm3
...................ucccugagacccuaacuuguU........................................................................  48    1  Fm3
...................ucccugagaccUuaacuugug........................................................................  8     1  Fm3
...................ucAcugagacccuaacuugug........................................................................  5     1  Fm3
...................ucccuNagacccuaacuugug........................................................................  1     1  Fm3
...................ucccugagacAcuaacuugug........................................................................  7     1  Fm3
...................ucccugagaUccuaacuugug........................................................................  16    1  Fm3
...................ucccugagacccuaacCugug........................................................................  16    1  Fm3
...................Ncccugagacccuaacuugug........................................................................  5     1  Fm3
...................ucccCgagacccuaacuugug........................................................................  1     1  Fm3
...................ucccugagacccuaacuuAug........................................................................  1     1  Fm3
...................ucccugagacccGaacuugug........................................................................  3     1  Fm3
...................ucccugagacccAaacuugug........................................................................  1     1  Fm3
...................ucUcugagacccuaacuugug........................................................................  3     1  Fm3
...................uAccugagacccuaacuugug........................................................................  1     1  Fm3
...................uccAugagacccuaacuugug........................................................................  4     1  Fm3
...................uccGugagacccuaacuuguga.......................................................................  2     1  Fm3
```

```
                   Mature                              Star
cguggaggcuuccuccccuucccugagacccuaacuugugauguaauaaaauucccgguuauggucuugggaauagggugguugcuauggcaacggaauacgacuaucacc
...................ucccugGgacccuaacuuguga......................................................................    2      1    Fm3
...................ucccugagacccuaacuAguga......................................................................    178    1    Fm3
...................ucccugagacccuaUcuuguga......................................................................    10     1    Fm3
...................uUccugagacccuaacuuguga......................................................................    13     1    Fm3
...................ucccugaCacccuaacuuguga......................................................................    1      1    Fm3
...................ucccugagacAcuaacuuguga......................................................................    71     1    Fm3
...................Acccugagacccuaacuuguga......................................................................    5      1    Fm3
...................ucccugagacccuaCcuuguga......................................................................    1      1    Fm3
...................ucccugagacccuaacGuguga......................................................................    91     1    Fm3
...................ucccCgagacccuaacuuguga......................................................................    3      1    Fm3
...................ucccugaAacccuaacuuguga......................................................................    8      1    Fm3
...................ucccugagacccCaacuuguga......................................................................    2      1    Fm3
...................ucccugagacccuaaUuuguga......................................................................    4      1    Fm3
...................Ccccugagacccuaacuuguga......................................................................    10     1    Fm3
...................ucccugagaAccuaacuuguga......................................................................    8      1    Fm3
...................ucccugagacccGaacuuguga......................................................................    5      1    Fm3
...................ucccugagacccuaacCuguga......................................................................    101    1    Fm3
...................uAccugagacccuaacuuguga......................................................................    26     1    Fm3
...................ucccugagacccuaacuCguga......................................................................    235    1    Fm3
...................ucccuCagacccuaacuuguga......................................................................    4      1    Fm3
...................ucccugaUacccuaacuuguga......................................................................    11     1    Fm3
...................ucccugagacUcuaacuuguga......................................................................    603    1    Fm3
...................ucccugagacccuaacuGguga......................................................................    140    1    Fm3
...................Ncccugagacccuaacuuguga......................................................................    20     1    Fm3
...................ucccuUagacccuaacuuguga......................................................................    6      1    Fm3
...................ucccugNgacccuaacuuguga......................................................................    2      1    Fm3
...................ucGcugagacccuaacuuguga......................................................................    13     1    Fm3
...................ucccugagCcccuaacuuguga......................................................................    4      1    Fm3
...................ucccugagacccuaacuugugU......................................................................    868    1    Fm3
...................ucccugagacccAaacuuguga......................................................................    4      1    Fm3
...................ucccGgagacccuaacuuguga......................................................................    1      1    Fm3
...................ucccuAagacccuaacuuguga......................................................................    6      1    Fm3
...................ucccugUgacccuaacuuguga......................................................................    10     1    Fm3
...................ucccugagacccuaaGuuguga......................................................................    2      1    Fm3
...................ucccugagacccuaacuuguAa......................................................................    12     1    Fm3
...................ucccugagUcccuaacuuguga......................................................................    33     1    Fm3
...................ucccugagacGcuaacuuguga......................................................................    46     1    Fm3
...................ucAcugagacccuaacuuguga......................................................................    20     1    Fm3
...................ucccugagacccuaGcuuguga......................................................................    5      1    Fm3
...................ucccugCgacccuaacuuguga......................................................................    2      1    Fm3
...................ucccugagacccuaacuuUuga......................................................................    7      1    Fm3
...................ucccugagacccuaacuugAga......................................................................    6      1    Fm3
...................ucccugagaccGuaacuuguga......................................................................    3      1    Fm3
...................ucccugagacccuaacuugugG......................................................................    22     1    Fm3
...................ucccugagacccuaacuuCuga......................................................................    1      1    Fm3
...................ucccugagaGccuaacuuguga......................................................................    3      1    Fm3
...................ucccugagaccUuaacuuguga......................................................................    18     1    Fm3
...................uccAugagacccuaacuuguga......................................................................    16     1    Fm3
...................ucccugagGcccuaacuuguga......................................................................    3      1    Fm3
...................ucccugagacccuaaAuuguga......................................................................    2      1    Fm3
...................ucccugagacccuaacuugCga......................................................................    2      1    Fm3
...................ucUcugagacccuaacuuguga......................................................................    4      1    Fm3
...................ucccugagacccuaacuuguga......................................................................    36202  0    Fm3
...................ucccugagaccAuaacuuguga......................................................................    15     1    Fm3
...................uccUugagacccuaacuuguga......................................................................    3      1    Fm3
...................ucccuNagacccuaacuuguga......................................................................    6      1    Fm3
...................ucccugagacccuaacAuguga......................................................................    86     1    Fm3
...................ucccugagacccuaacuuAuga......................................................................    3      1    Fm3
...................uGccugagacccuaacuuguga......................................................................    11     1    Fm3
...................ucccugagacccuaacuugGga......................................................................    4      1    Fm3
...................Gcccugagacccuaacuuguga......................................................................    2      1    Fm3
...................ucccugagacccuaacuuguUa......................................................................    14     1    Fm3
...................ucccugagacccuaacuugugC......................................................................    4      1    Fm3
...................ucccugagacccuGacuuguga......................................................................    4      1    Fm3
...................ucccugagacccuaacuuguCa......................................................................    4      1    Fm3
...................ucccugagacccuUacuuguga......................................................................    1      1    Fm3
...................ucccugagaUccuaacuuguga......................................................................    27     1    Fm3
...................ucccugagacccuaacuGgugau.....................................................................    3      1    Fm3
...................ucGcugagacccuaacuugugau.....................................................................    1      1    Fm3
...................ucccugagacccuaacuugugaA.....................................................................    2537   1    Fm3
```

Mature / Star			
cguggaggcuuccuccccuucccugagacccuaacuugugauguaauaaaauucccgguuauggucuugggaauagggugguugcuauggcaacggaauacgacuaucacc			
...................uGccugagacccuaacuugugau...	1	1	Fm3
...................ucccugaUacccuaacuugugau...	1	1	Fm3
...................ucccugagacccuaacuugugaG...	316	1	Fm3
...................ucccugagacUcuaacuugugau...	2	1	Fm3
...................ucccugagacccuaacuAgugau...	4	1	Fm3
...................ucccugagacccuaacuugugau...	278	0	Fm3
...................ucccugagacccuaacuugugaC...	2	1	Fm3
...................ucccugagaccUuaacuugugau...	2	1	Fm3
...................ucccugagacccuaacGugugau...	2	1	Fm3
...................ucccugagacccuaacAugugau...	2	1	Fm3
...................ucccugagacccuaacuugugUu...	13	1	Fm3
...................ucccugagacccuaacuugugaug..	13	0	Fm3
...................ucUcugagacccuaacuugugaug..	1	1	Fm3
...................ucccugagacccuaacuugugauC..	2	1	Fm3
...................ucccugagacccuaacuugugauA..	4	1	Fm3
...................ucccugagacccuaacuAgugaug..	1	1	Fm3
...................ucccugagacccuaacuugugaAg..	18	1	Fm3
...................ucccugagacccuaacuugugauU..	31	1	Fm3
...................ucccugagacccuaacuugugauUu...	1	1	Fm3
...................ucccugagacccuaacuugugaugua..	1	0	Fm3
....................cccugagacccuaacuugu..	2	0	Fm3
....................Uccugagacccuaacuugu..	1	1	Fm3
....................cccugagacccuaacuugug...	1	0	Fm3
....................cccugagacUcuaacuuguga..	4	1	Fm3
....................cccugagacccuaacuuAuga..	1	1	Fm3
....................cccugagacccuaacuuguga..	99	0	Fm3
....................Uccugagacccuaacuuguga..	10	1	Fm3
....................cccugaUacccuaacuuguga..	1	1	Fm3
....................cccugagacccuaacuugugaA...	13	1	Fm3
.....................ccugagacccuaacuugu..	3	0	Fm3
.....................ccugagacccuaacuugug...	7	0	Fm3
.....................ccugagacccuaacuuguga..	28	0	Fm3
.....................ccugagacccuaacuugugG..	1	1	Fm3
.....................ccugagacccuaaAuuguga..	2	1	Fm3
.....................ccugagacccuaacuugugaA...	4	1	Fm3
......................cugagacccuaacuuguga..	1	0	Fm3
.......................ugagacccuaacuuguga..	24	0	Fm3
.......................ugagacccuaacuugugU..	3	1	Fm3
.......................ugagacccuaacuugugaG...	2	1	Fm3
.................................acuugugauguaauaaaauuc...	1	0	Fm3
....cAgguuauggucuugggaa......................................	1	1	Fm3
....ccgguuauggucuugggaa......................................	4	0	Fm3
....ccgguuauggucuugggaau.....................................	2	0	Fm3
....ccgguuauggucuugggaaua....................................	1	0	Fm3
....cgguuauggucuugggaaua....................................	1	0	Fm3
....uuauggucuugggaauagA..................................	1	1	Fm3
